# Anaplastic Lymphoma Kinase Positive Mesenteric Inflammatory Myofibroblastic Tumor in Adult Woman

**DOI:** 10.7759/cureus.24422

**Published:** 2022-04-23

**Authors:** Christina S Lee, Jason S Kim, Rosemarie Rodriguez, Robert W Krell

**Affiliations:** 1 General Surgery, San Antonio Military Medical Center, San Antonio, USA; 2 Radiology, Brooke Army Medical Center, San Antonio, USA; 3 Pathology, Brooke Army Medical Center, San Antonio, USA; 4 General Surgery, Brooke Army Medical Center, San Antonio, USA

**Keywords:** soft-tissue tumor, intra-abdominal soft tissue tumor, anaplastic lymphoma kinase, imt, inflammatory myofibroblastic tumor (imt)

## Abstract

Inflammatory myofibroblastic tumors (IMTs) are rare mesenchymal neoplasms containing spindle cells and inflammatory components that can be locally aggressive. They have unclear biological behavior and may recur after resection.

A 31-year-old woman presented with three months of cough, fatigue, weight loss, abdominal pain, anemia, and elevated inflammatory markers. CT showed a large well-circumscribed enhancing mass in the right colic mesentery. The patient underwent a laparoscopic right colectomy. Pathologic review showed fascicular spindle cells with admixed chronic inflammatory cells. Cells stained diffusely positive for SMA and anaplastic lymphoma kinase (ALK), diagnostic of an IMT. Post-operatively, the patient reported symptom resolution and had normalization of lab values. She remains disease-free at 20 months.

IMT is rare in adults, accounting for 0.7%-1.0% of lung tumors. Up to 30% of patients present with elevated inflammatory markers. On imaging, IMTs are soft tissue masses with variable enhancement and fibrosis, often suspected to be malignant neoplasms. Up to 80% of IMTs are driven by altered tyrosine kinase signaling and half of IMTs express ALK, which may be treated in unresectable/recurrent cases using ALK-inhibitors. IMT may recur in 10%-15% of patients. The roles of adjuvant treatments are unclear given the rarity and unpredictable biological behavior. Long-term follow-up with regular radiologic and laboratory surveillance is recommended given possible local recurrence. IMTs are best managed in a multidisciplinary setting given their unpredictable nature. Surgery is the mainstay of IMT treatment with long-term control expected in >80% of adult patients.

## Introduction

Inflammatory myofibroblastic tumors (IMTs) are rare mesenchymal neoplasms with uncertain malignant potential and an estimated incidence of 150-200 new cases per year [1-3]. They were first described as pulmonary tumors, and extrapulmonary sites are extremely rare [1]. On histology, IMTs show spindle cell proliferation in a myxoid to collagenous stroma with prominent inflammatory infiltrates such as plasma cells and lymphocytes.

Patients with IMT may be asymptomatic or present with local symptoms such as pain, as well as signs of systemic inflammation such as fever and/or elevated acute phase reactants in about 15%-30% of cases [4]. The mainstay of treatment for localized IMT is complete surgical resection.

Given their rarity, the biological behavior of IMT remains poorly understood. Because up to 15%-30% recur after resection or metastasize distally, IMT is described as having an “intermediate (rarely metastasizing) biological potential” according to the World Health Organization [5]. In non-resectable diseases, different treatment modalities have been reported including corticosteroids, chemotherapy, non-steroidal anti-inflammatory drugs, and radiation therapy [6]. 

IMT requires a high index of suspicion for diagnosis and close coordination between surgical, medical, and pathology specialists with soft tissue neoplasm expertise. Herein, we report a 31-year-old woman who presented with a chronic cough and abdominal pain diagnosed with mesenteric IMT on immunohistochemistry after a right colectomy.

This article was previously presented as a poster at the Annual South Texas Chapter of the American College of Surgeons in Houston, TX, USA, on February 17-19, 2022.

## Case presentation

A 31-year-old Caucasian woman presented to the emergency department with sharp abdominal pain occasioned after a coughing fit. History revealed three months of dry cough, fatigue, chills, night sweats, and 25-pound weight loss prior to her presentation. She denied symptoms of bowel obstruction or gastrointestinal bleed. She underwent colonoscopy six years prior to presentation for chronic abdominal discomfort, which did not reveal any colonic pathology. The patient reported a family history of a maternal grandfather with prostate cancer. On physical exam, she had a palpable mobile right abdominal mass without palpable cervical, axillary, or inguinal lymphadenopathy.

**Figure 1:**
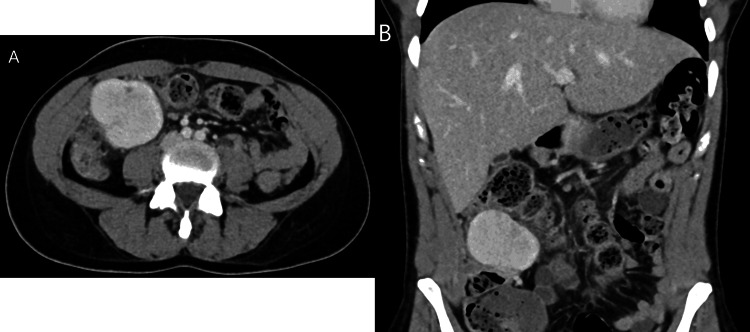
Axial (A) and coronal (B) contrast-enhanced CT images of the abdomen demonstrate a circumscribed avidly enhancing mass arising from the mesentery near the hepatic flexure.

Laboratory workup was significant for anemia (hemoglobin 6.8g/dL) and elevated inflammatory markers including elevated erythrocyte sedimentation rate (ESR) at >120 mm/hour (ref: <15-20 mm/hour), C-reactive protein (CRP) at 20.8 mg/dL (ref: 0-0.49 mg/dL), ferritin 564 ng/mL (ref: 15-150 ng/mL), and thrombocytosis 726 x 10^3^/µL. Additional laboratory workups including lactate dehydrogenase, serum uric acid, human immunodeficiency virus serologies, chronic hepatitis serology panel, and serum protein electrophoresis were all normal. COVID-19 polymerase chain reaction (PCR) test was negative. Computed tomography (CT) scan of the abdomen and pelvis revealed a 6.0 x 4.0 x 5.7cm mass within the right colon mesentery (Figures 1A, 1B). The mass demonstrated peripheral inflammatory changes, patchy arterial phase enhancement, and avid venous phase enhancement. CT chest was normal.

**Figure 2:**
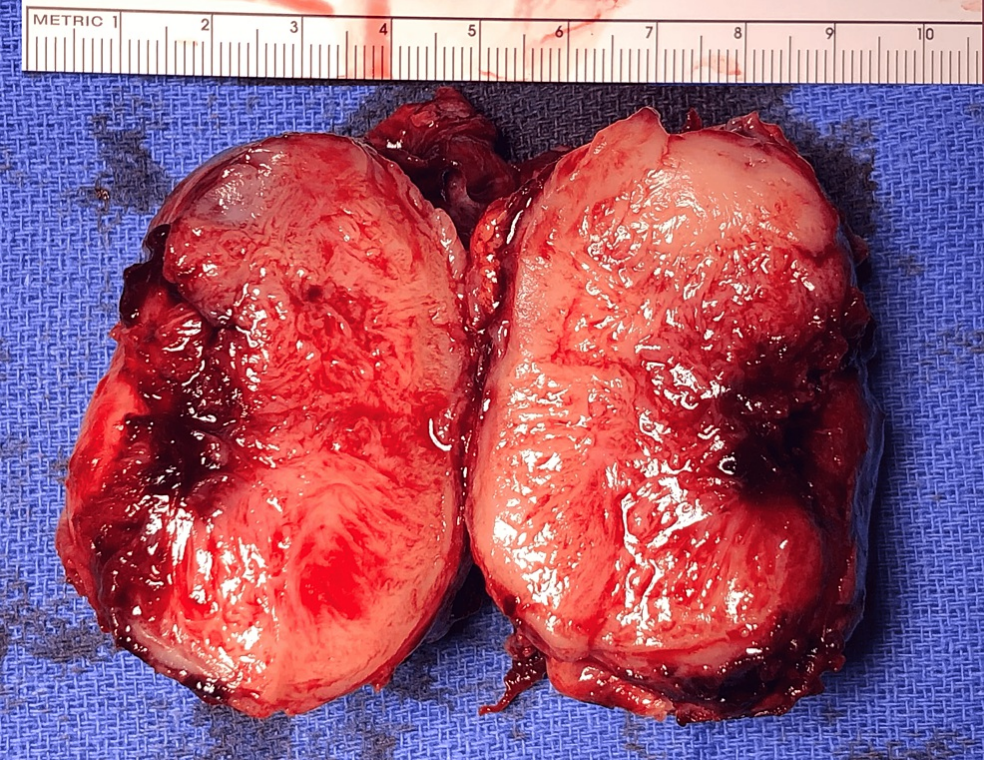
Operative specimen shows well-circumscribed mass measuring 4.5 x 6.5 cm with firm, rubbery white surface when bisected.

The initial differential diagnosis included: lymphoma, Castleman’s disease, Kaposi’s sarcoma, gastrointestinal stromal tumor, soft tissue sarcoma, nerve sheath tumor, and neuroendocrine tumor. We advised resection for symptom palliation and definitive diagnosis. She underwent diagnostic laparoscopy with laparoscopic mass resection. During the operation, the mass arose from the mesentery of the hepatic flexure of the colon and had an inflammatory capsule in the surrounding mesentery with clearly uninvolved anterior and posterior margins. The ascending colon and hepatic flexure were normal but closely involved. The mass was dissected free from surrounding mesentery and colon and sent for a frozen section. Figure 2 shows the operative specimen. The frozen section showed spindle cell neoplasm, so completion laparoscopic right colectomy was performed to ensure adequate margin clearance.

**Figure 3:**
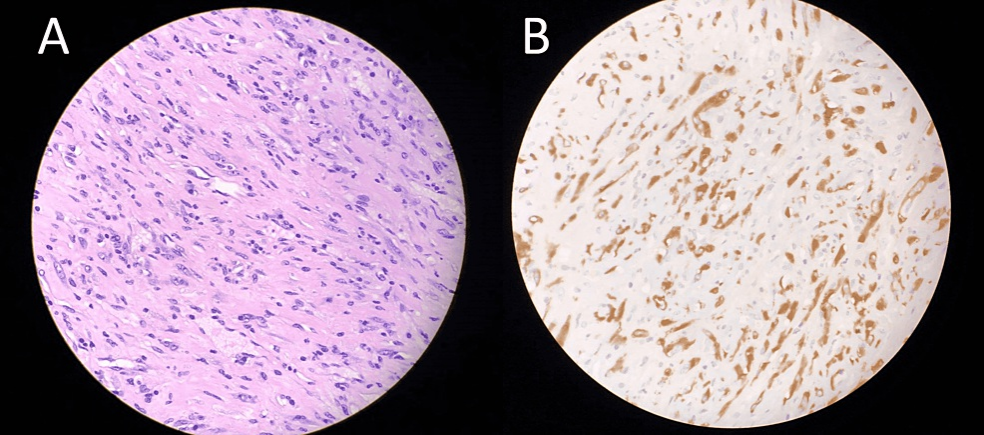
(A) Hematoxylin and eosin stain showing fascicular proliferation of spindle cells with palely eosinophilic cytoplasm and plump mildly typical vesicular or more tapering nuclei. (B) Strong positive stain ALK expression on immunohistochemistry. Not shown: Negative stains for AE1/AE3, Lu-5, EMA, CK7, CK20, CD45, S100, PAX8, GATA3, CD34, myogenin, CD117, Dog-1, CDX2, SOX10, HMB45, p63, CD15, CD30, CD21, CD23, calretinin, and EBER ISH.

The patient had an uneventful surgical recovery and was discharged home on postoperative day 3. Pathologic analysis showed a fascicular proliferation of spindle cells having palely eosinophilic cytoplasm and plump mildly atypical vesicular or more tapering nuclei (Figures 3A, 3B). There were numerous admixed chronic inflammatory cells. There was no pleomorphism and no atypical mitoses seen. On immunohistochemistry analysis, the neoplastic cells were diffusely positive for smooth muscle actin (SMA), scattered desmin positivity, and negative for AE1/AE3, Lu-5, EMA, CK7, CK20, CD45, S100, PAX8, GATA3, CD34, myogenin, CD117, Dog-1, CDX2, SOX10, HMB45, p63, CD15, CD30, CD21, CD23, calretinin, and EBER ISH. There was strong and diffuse cytoplasmic positivity for ALK. The appearance and staining pattern was consistent with IMT. Next-generation sequencing (Foundation One ®) Heme genomic profile showed TFG-ALK fusion and a FISH break-apart probe showed that 47% of tumor nuclei carried the rearrangement of the ALK gene at 2p23.2, which is also consistent with IMT. Margins were widely clear of tumors.

At her two-week postop visit, her anemia and inflammatory markers had normalized. At six-month follow-up, the patient had completely recovered and had no abdominal symptoms. Surveillance CT at six and 12 months showed no recurrence. The patient remains without evidence of recurrence at 20 months since the surgery. She will continue biannual surveillance imaging and laboratory workup.

## Discussion

IMT is a rare neoplasm initially described as pediatric lung and pleural tumors. IMT’s hallmark features are a proliferation of myofibroblastic spindle cells with variable inflammatory components such as lymphocytes or eosinophils. Genetic studies of IMTs have shown chromosomal aberrations suggesting clonal origin, therefore making it a true neoplasm [7].

While IMT accounts for up to 20% of primary lung tumors in children, it is exceptionally rare in adults at about 0.7%-1.0% of lung tumors [8]. In one of the largest case series of 84 patients with extra-pulmonary IMT managed at two referral centers over 16 years, Coffin et al. showed 36 (43%) cases arose from the mesentery and omentum [9]. Presenting signs and symptoms differ based on the location of the lesion. Patients with intraabdominal IMT may present with nonspecific symptoms such as pain, nausea, and vomiting as well as systemic symptoms such as fever, chills, fatigue, and weight loss. Up to 30% of patients with IMT present with signs of systemic inflammation with elevated inflammatory markers such as erythrocyte sedimentation rate, anemia, thrombocytosis, and hypergammaglobulinemia [5]. Why IMT is associated with systemic inflammation is poorly understood. As seen in the patient presented here, most patients have a resolution of symptoms and normalization of the laboratory values following surgical resection [10].

IMTs have varied and nonspecific imaging characteristics [11-13]. In general, IMTs present as a soft tissue mass with variable enhancement and fibrosis, often initially suspected to be malignant neoplasms [11]. IMTs of the mesentery often demonstrate imaging features that overlap with various inflammatory or neoplastic conditions such as sclerosing mesenteritis, lymphoma, and sarcoma. On CT, mesenteric IMTs present with variable enhancement ranging from no enhancement, heterogeneous enhancement, or peripheral enhancement depending on the degree of fibrosis [14]. Larger tumors may show central necrosis or calcification [13]. Mesenteric IMTs on ultrasound have been described as well-defined or ill-defined, heterogeneous, hypoechoic, or hyperechoic solid masses with internal complexity and vascularity [15,16]. On MR imaging, IMTs are typically T1-hypointense and T2-hypointense with variable internal T1 and T2-hypointense fibrotic stroma with heterogeneous gadolinium enhancement [12,16]. The variable imaging findings are reflective of the histological variability of the tumor and are often a cause of diagnostic confusion.

 Pathologic diagnosis of IMT requires careful consideration of the differentiation from a variety of spindle cell neoplasms including desmoid tumors, gastrointestinal stromal tumors, peripheral nerve sheath tumors, leiomyomata, or leiomyosarcoma. IMT generally stains very strongly for vimentin. Additionally, IMT can show variable expression of SMA, muscle-specific actin, and desmin [17]. Keratin stain may be positive in about 40%-70%. About 50% of IMT shows diffuse positive cytoplasmic staining using monoclonal antibodies targeting anaplastic lymphoma kinase (ALK). ALK is a tyrosine kinase of the insulin growth factor receptor superfamily. About 40% of IMTs have a characteristic translocation in chromosome 2p23 which activates the expression of p80 and ALK1 [18]. Next-generation sequencing studies show many ALK-negative tumors harbor an activating tyrosine kinase fusion involving the ROS1 or PDGFRβ genes [19]. Diagnosis of ALK-negative IMT is a diagnosis of exclusion.

The mainstay of treatment for IMT is R0 surgical resection as long as technically and anatomically feasible. Even in the setting of incomplete resection, long-term outcomes are favorable; progression or local recurrence occurs in 10%-15% of patients who undergo incomplete resection [6,9]. Metastases occur in 5% or fewer patients and are exceptionally rare in adult patients [6]. There are no cumulative data beyond case reports regarding the efficacy of adjuvant systemic or radiation therapies given IMT's rarity and uncertain biologic behavior.

In unresectable/metastatic cases, systemic treatments are generally recommended. A case series of 16 patients with ALK-positive unresectable IMT showed a complete or partial response rate of 75% to ALK inhibitors, similar to that seen in a Children’s Oncology Group study of 14 children, showing an 86% response rate [20]. The use of targeted ALK inhibitors may be considered for ALK-positive unresectable IMTs.

Long-term follow-up of all patients with IMT is recommended given the unclear biology of these tumors and unpredictable recurrence risk. There are no reliable predictors of the 10%-15% of patients who will experience recurrence. Most experts recommend regular surveillance of radiologic and laboratory workup for IMT patients who undergo resection. There are no guidelines regarding the frequency of follow-up in these rare tumors; most experience is extrapolated from the sarcoma field and many experts advise surveillance imaging every six months for the first three to five years [21].

## Conclusions

IMTs are exceptionally rare tumors with ambiguous presentation and unclear behavior. Their diagnosis and treatment warrant multi-disciplinary discussion and cooperation among those with expertise in soft tissue tumors including surgeons, medical specialists, radiologists, and pathologists for successful management. While 85%-90% of patients remain without recurrence after surgical resection, we recommend continued surveillance with imaging and laboratory studies.
